# The impact of molecular profile on the lymphatic spread pattern in stage III colon cancer

**DOI:** 10.1111/cas.14819

**Published:** 2021-02-18

**Authors:** Jihyung Song, Kozo Kataoka, Takeshi Yamada, Manabu Shiozawa, Tomohiro Sonoyama, Naohito Beppu, Koji Ueda, Sho Kuriyama, Akiyoshi Kanazawa, Masataka Ikeda, Wim Ceelen

**Affiliations:** ^1^ Department of Gastroenterological Surgery Division of Lower GI Hyogo College of Medicine Nishinomiya Japan; ^2^ Department of Gastrointestinal Surgery Nippon Medical School Tokyo Japan; ^3^ Department of Gastrointestinal Surgery Kanagawa Cancer Center Yokohama Japan; ^4^ Department of Pharmacy Shimane Prefectural Central Hospital Izumo Japan; ^5^ Department of Surgery Shimane Prefectural Central Hospital Izumo Japan; ^6^ Department of GI Surgery Ghent University Hospital, and Cancer Research Institute Ghent (CRIG) Ghent University Ghent Belgium

**Keywords:** BRAS, colon cancer, KRAS, lymphatic spread pattern, MSI

## Abstract

The anatomical spread of lymph node (LN) metastasis is of practical importance in the surgical management of colon cancer (CC). We examined the effect of KRAS, BRAF, and microsatellite instability (MSI) on LN count and anatomical spread pattern in stage III CC. We determined KRAS, BRAF, and MSI status from stage III CC patients. Biomarker status was correlated with LN count and anatomical spread pattern, which was classified as sequential or skipped. Relapse‐free survival (RFS) was estimated using Kaplan‐Meier method, and correlations were assessed using log‐rank and Cox regression analyses. We analyzed 369 stage III CC patients. The proportion of KRAS mutant (mt), BRAF mt, and MSI‐high (H) were 44.2% (163/344), 6.8% (25/344), and 6.8% (25/344), respectively. The mean number of metastatic LN was higher in microsatellite‐stable (MSS) compared with MSI patients (3.5 vs. 2.7, *P* = .0406), although no differences were observed in accordance with KRAS or BRAF status. Interestingly, patients with BRAF mt and MSI‐H were less likely to harbor skipped metastatic LN (9.3% vs 20% and 4% vs 10.5% compared with BRAF wild‐type (wt) and MSS, respectively), but KRAS status did not predict anatomical spread pattern. Patients with KRAS wt and MSI‐H showed superior RFS compared with KRAS mt and MSS patients, respectively, whereas BRAF status did not affect RFS. Differences exist in the anatomical pattern of invaded LN in accordance with the molecular status of stage III CC. Patients with MSI‐H CC have less invaded and skipped LN, suggesting that a tailored surgical approach is possible.

## Graphical abstract

The association of KRAS/BRAF/MSI with invaded lymphatic spread pattern in colon cancer was assessed. The anatomical pattern of invaded LN varies in accordance with the molecular status, which may affect the outcome in stage III colon cancer.

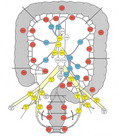

**Abbreviations**
CC: colon cancer
CME: complete mesocolic excision
CMS: consensus molecular subtypes
CRC: colorectal cancer
CVL: central venous ligation
LN: lymph node
MSI: microsatellite instability
MSS: microsatellite‐stable
mt: mutant
OSNA: one‐step nucleic acid amplification
RFS: Relapse‐free survival

## 1 INTRODUCTION

Colorectal cancer (CRC) is the third most common cancer worldwide, and accounts for approximately 1.7 million cases and 700 000 deaths per year.^1^ Over the past few years, the treatment of metastatic CRC has been personalized based on molecular and biological factors. In patients with RAS wild‐type (wt) CRC, anti‐epidermal growth factor receptor (anti‐EGFR) therapy has been used^2^, ^3^ and immune checkpoint inhibitors such as anti‐PD‐1 and anti‐PDL‐1 antibodies are effective in microsatellite instable (MSI‐high) patients.^4^ Recently, the efficacy of encorafenib, binimetinib, and cetuximab for BRAF mutant (mt) CRC was confirmed in a phase III trial.^5^ However, current surgical treatment of stage III CRC does not incorporate the status of these molecular biomarkers.

Colon cancer spreads along hematogenous and lymphatic pathways, but the exact clinical relevance and anatomical pathways of lymphatic spread in CC are incompletely elucidated.^6^, ^7^, ^8^, ^9^ We previously reported that the anatomical pattern of lymphatic spread, and its prognostic implications, differed between right and left colon cancer.^8^, ^9^ Whether molecular biomarkers such as KRAS, BRAF and MSI status correlate with the extent and anatomical pattern of LN metastasis in stage III CC is currently unknown.^10^, ^11^ Insight into these relationships may allow tailoring of the surgical approach, and the extent of lymphadenectomy, based on the molecular status of the tumor.

Here, we investigated whether KRAS, BRAF, and MSI status were related to the extent of nodal involvement, the anatomical pattern of lymphatic spread, and RFS in a cohort of stage III CC patients who underwent extensive lymphadenectomy.

## 2 METHODS

### 2.1 Patient selection

Data from pathological stage III CC patients from 4 specialized centers (Hyogo College of Medicine, Nippon Medical University Hospital, Kanagawa Cancer Center and Shimane Prefectural Central Hospital) who were treated between 2012 and 2018 were collected retrospectively. The patients were all treated with extensive lymphadenectomy (Japanese D3 dissection) and achieved a pathological R0 resection. The 7th edition of the UICC TNM classification was used.^12^ Patients treated with neoadjuvant treatment and patients with rectal cancer were excluded. This retrospective study was approved by the Institutional Review Board of Hyogo College of medicine, Japan (N0. 3445).

### 2.2 Surgical treatment/chemotherapy

Open or laparoscopic colonic resection with Japanese D3 LN dissection was performed in all patients. For right‐sided cancers, central vascular ligation was performed, removing draining LNs (stations 203, 213, and 223) along the superior mesenteric vein. For left‐sided cancer, either high ligation of the inferior mesenteric artery was performed, with removal of LNs at station 253, or the left colic artery was preserved, and the superior rectal artery divided at its origin. This technique is theoretically similar to CME with CVL.^13^, ^14^ Adjuvant therapy was administered based on local practice.

### 2.3 Definition of LN spreading pattern and L level

**FIGURE 1:**
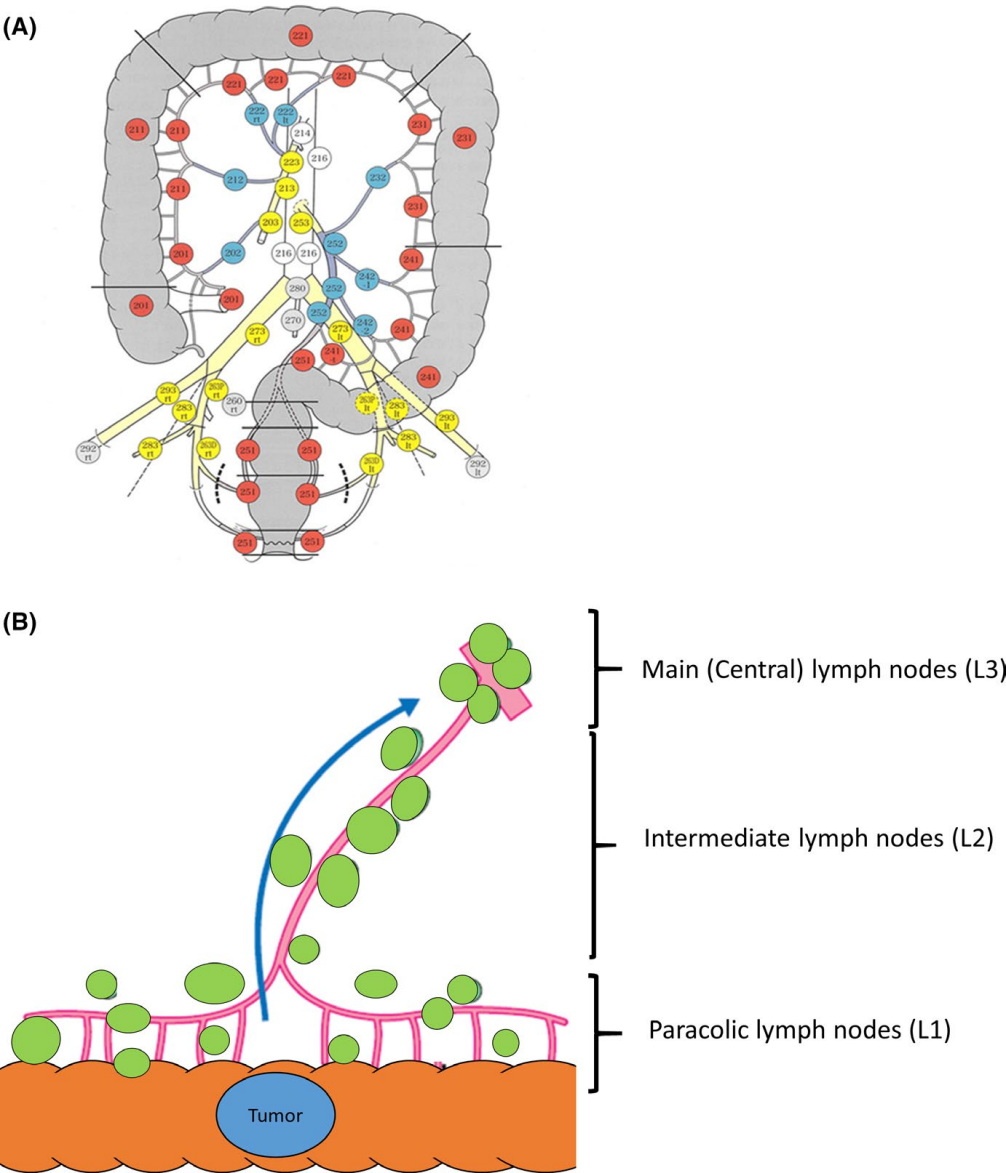
A, Anatomical LN stations in accordance with the JSCCR. B, Route of the lymphatic metastasis is described in this schema. The Halsted model (green dotted arrows) assumes stepwise progression from the primary tumor over L1→L2→L3 nodes and ultimately to distant organs. The Fisher model (yellow dotted lines), conversely, the presupposes that spread of metastatic tumor cells occurs early and to paracolic nodes, central nodes, or metastatic sites in a random manner. T, tumor; M, metastasis

According to the Japanese Society for Cancer of the Colon and Rectum (JSCCR), invaded LNs were classified into 3 groups, as carried out in our previous studies: ^8^, ^9^ L1 (paracolic), L2 (intermediate) and L3 (main or central) (Figure 1). The anatomical pattern of metastatic LN spread was classified as *sequential* when a positive (cancer invaded) LN occurred only when all previous LN stations were positive, and as *skipped* whenever a positive LN was identified with 1 or more previous nodal stations (L1 and/or L2) negative.

### 2.4 Mutational analysis of KRAS and BRAF, and assessment of MSI

Colorectal cancer specimens and the adjacent normal tissues for comparison were retrospectively collected from patients who had undergone surgery. All protocols were approved by the ethics committee of Hyogo College of Medicine as RINHI‐0120 and all patients provided written informed consent. These specimens were stored in RNAlater at −80°C before use. Genomic DNA was extracted using the QIAamp DNA mini kit (Qiagen, Venlo, Netherlands). Genomic DNA was used to evaluate their microsatellite instability (MSI) status. The mononucleotide microsatellite markers BAT‐25, BAT‐26, NR21, NR22, and NR24 were used for evaluation as previously described.^15^ We also analyzed the mutations of KRAS codon 12, KRAS codon 13, and BRAF codon 600 using Sanger sequencing. All analyses were performed at the Hyogo College of Medicine and Nippon Medical School Hospital.

### 2.5 Statistical analysis

The chi‐square or Fisher exact test was used to evaluate differences between proportions, and Student *t* test or Mann‐Whitney *U* test was used to evaluate differences between means, as appropriate. RFS were estimated from the date of surgery until recurrence or death from any cause. The Kaplan‐Meier method and log‐rank test were used for survival analysis. Multivariate Cox proportional hazards regression was used to identify independent prognostic variables. The presence of pathological lymphatic invasion and venous invasion was classified in accordance with the JSCCR system: 0; no invasion, 1; mild invasion, 2; moderate invasion, and 3; severe invasion.^16^ Pathological lymphatic and venous invasions were analyzed as categorical covariates. All analyses were performed with IBM® SPSS® Statistics, version 25. *P*‐values were reported and interpreted in accordance with recent guidelines from the American Statistical Association.^17^

## 3 RESULTS

In total, data from 369 stage III CC patients were collected and analyzed. The median follow‐up time was 53 mo. Patient characteristics are shown in Table 1. The proportion of KRAS mt, BRAF mt, and MSI‐H patients was 44.2% (163/206), 6.8% (25/344), and 6.8% (25/344), respectively. The proportion of BRAF mt and MSI‐H in each anatomical location was different from that of BRAF wt and MSI‐H, respectively (BRAF; *P* = .00735, MSI; *P* = .0306) but there was no difference regarding KRAS (*P* = .524). The mean number of *harvested* LN was lower in BRAF mt compared with BRAF wt patients (20.0 vs. 24.8; *P* = .0360), but the mean number of *invaded* LN did not differ based on BRAF status. Conversely, MSI status did not affect the number of *harvested* LN (23.3 vs. 24.6 in MSI‐H and MSS, respectively; *P* = .626), but the mean number of *invaded* LN was higher in MSS patients (3.5 vs 2.7; *P* = .0410). The incidence of central LN positive (L3) disease was similar regardless of biomarker status. Adjuvant therapy was given to 260/369 patients (70.5%); 90 of these patients (24.4%) received doublet chemotherapy such as mFOLFOX6 or CapOX.

**TABLE 1:** Patient characteristics

	Total	KRAS			BRAF			MSI		
	N = 369	Wild‐type N = 206	Mutant N = 163	*P*	Wild‐type N = 344	Mutant N = 25	*P*	MSS N = 344	MSI‐H N = 25	*P*
Gender (%)										
Male	188 (51.0)	110 (53.4)	71 (43.6)	.0745	170 (49.4)	11 (44.0)	.681	173 (50.3)	8 (32.0)	.0974
Female	181 (49.0)	96 (46.6)	92 (56.4)		174 (51.6)	14 (56.0)		171 (49.7)	17 (68.0)	
Age										
Mean	68.7	68.8	68.6	.859	68.3	74.0	**.0190**	68.4	72.1	.196
Tumor size (mm)										
Mean	47.0	46.7	47.4	.851	46.9	48.3	.339	46.9	48.5	.205
Anatomical location (%)										
C	41 (11.1)	18 (8.7)	23 (14.1)	.524	34 (9.8)	7 (28.0)	**.00735**	36 (10.4)	5 (20.0)	**.0306**
A	76 (20.5)	39 (18.9)	37 (22.7)		68 (19.7)	8 (32.0)		67 (19.4)	9 (36.0)	
T	42 (11.3)	25 (12.1)	17 (10.4)		38 (11)	4 (16.0)		37 (10.7)	5 (20.0)	
D	22 (5.9)	13 (6.3)	9 (5.5)		20 (5.8)	2 (8.0)		21 (6.1)	1 (4.0)	
S	112 (30.3)	67 (32.5)	45 (27.6)		109 (31.6)	3 (12.0)		108 (31.3)	4 (16.0)	
Rs	76 (20.9)	44 (27.8)	32 (19.7)		75 (22.1)	1 (4.0)		75 (22.1)	1 (4.0)	
T stage (%)										
T1	8 (2.2)	7 (3.3)	1 (0.6)	**.0399**	7 (2)	1 (4)	.745	8 (2.3)	0 (0)	.744
T2	19 (5.1)	13 (6.3)	6 (3.6)		17 (4.9)	2 (8)		17 (4.9)	2 (8)	
T3	222 (60.2)	129 (62.6)	93 (57)		209 (60.8)	13 (52)		206 (59.9)	16 (64)	
T4	120 (32.5)	57 (27.8)	63 (38.8)		111 (32.3)	9 (36)		113 (32.9)	7 (28)	
N stage (%)										
N1a	130 (35.2)	71 (34.4)	59 (36.1)	.778	125 (36.3)	5 (20)	0.103	120 (34.9)	10 (40)	.609
N1b	119 (32.2)	64 (31)	55 (33.7)		108 (31.4)	11 (44)		111 (32.3)	8 (32)	
N2a	75 (20.3)	43 (20.8)	32 (19.6)		67 (19.5)	8 (32)		69 (20.1)	6 (24)	
N2b	45 (12.3)	28 (13.8)	17 (10.6)		44 (12.5)	1 (4)		44 (12.7)	1 (4)	
Lymphatic invasion score (%)										
0	64 (17.3)	32 (15.5)	32 (19.6)	.496	63 (18.3)	1 (4)	**.00959**	62 (18)	2 (8)	**.0143**
1	178 (48.2)	97 (47)	81 (49.6)		170 (49.4)	8 (32)		170 (49.4)	8 (32)	
2	86 (23.3)	51 (24.7)	35 (21.4)		76 (22.1)	10 (40)		78 (22.7)	8 (32)	
3	41 (11.2)	26 (12.8)	15 (9.4)		35 (10.2)	6 (24)		34 (9.9)	7 (28)	
Venous invasion score (%)										
0	67 (18.2)	32 (15.5)	35 (21.4)	.438	64 (18.6)	3 (12)	.716	65 (18.9)	2 (8)	.111
1	170 (46.1)	99 (48)	71 (43.5)		156 (45.3)	14 (56)		153 (44)	17 (68)	
2	90 (24.4)	53 (25.7)	37 (22.6)		85 (25)	5 (20)		87 (25.3)	3 (12)	
3	42 (11.3)	22 (10.8)	20 (12.5)		39 (36.1)	3 (12)		39 (11.8)	3 (12)	
Number of LN (mean)										
Harvested	24.5	24.1	25.0	.536	24.8	20.0	**.0360**	24.6	23.3	.626
Positive	3.5	3.5	3.4	.825	3.5	3.3	.969	3.5	2.7	.0410
Positive anatomical LN level (L group; %)										
L1	245 (66.4)	128 (62.1)	117 (71.8)	.139	230 (66.9)	15 (60.0)	.175	226 (65.7)	19 (76.0)	.558
L2	89 (24.1)	55 (26.7)	34 (20.9)		84 (24.4)	5 (20.0)		85 (24.7)	4 (16.0)	
L3	35 (9.5)	23 (11.2)	12 (7.3%)		30 (8.7)	5 (20.0)		33 (9.6)	2 (8.0)	

Bold values indicate *P* < .05 is considered as significant.

The anatomical pattern of LN metastasis is shown in Table 2. Mutational status of KRAS did not affect the proportion of patients with skipped LN metastases (9.8% in KRAS mt and 10.2% skipped in KRAS wt). However, the proportion of BRAF mt and MSI‐H patients with a skipped lymphatic spread pattern tended to be lower compared with that of BRAF wt and MSS, (9.3% vs 20%; *P* = .0901 and 4% vs 10.5%, *P* = .259, respectively).

**TABLE 2:** The anatomical pattern of LN metastasis and molecular status

Invaded LN pattern (L1L2L3)	KRAS		BRAF		MSI	
	wt	mt	wt	mt	MSS	MSI‐H
Sequential (%)						
**+−−**	71.8	62.1	60.0	66.9	65.7	76.0
**++−**	14.1	20.4	12.0	18	18	16.0
**+++**	4.3	7.3	8.0	5.8	5.8	4.0
Total	90.2	89.8	80.0	90.7	89.5	96.0
Skipped (%)						
**−+−**	0.0	1.0	4.0	0.3	0.6	0.0
**+−+**	0.6	1.0	0.0	0.9	0.9	0.0
**−−+**	6.7	6.3	8.0	6.4	6.7	4.0
**−++**	2.5	1.9	8.0	1.7	2.3	0.0
Subtotal	9.8	10.2	20.0	9.3	10.5	4.0

**FIGURE 2:**
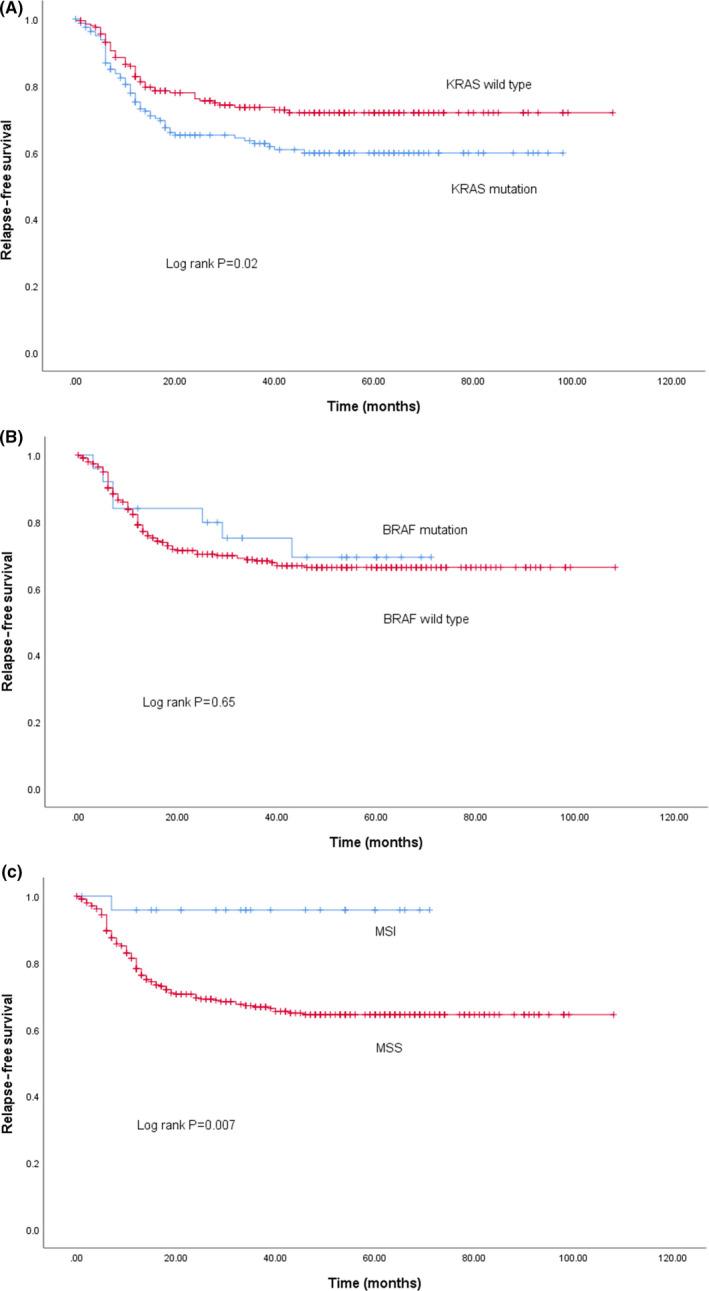
Unadjusted RFS in CC patients with; KRAS wt vs. KRAS mt (A; KRAS wt = red, KRAS mt = blue), BRAF wt vs. mt (B; BRAF wt = red, BRAF mt = blue), MSS vs. MSI‐H (MSS = red, MSI‐H = blue)

**FIGURE 3:**
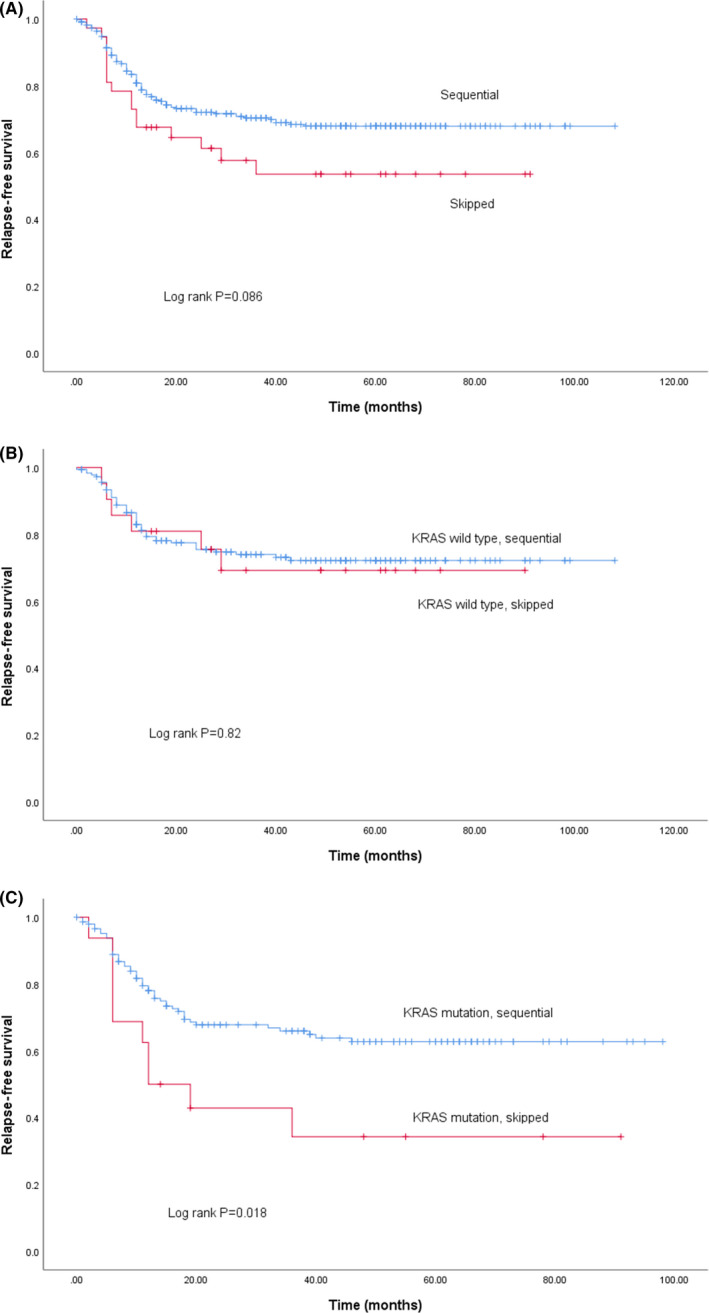
A, RFS in CC patients with sequential vs. skipped LN pattern (sequential = blue, skipped = red). B, RFS in KRAS wt CC patients with sequential vs. skipped LN pattern (sequential = blue, skipped = red). C, RFS in KRAS mt CC patients with sequential vs. skipped LN pattern (sequential = blue, skipped = red)

**FIGURE 4:**
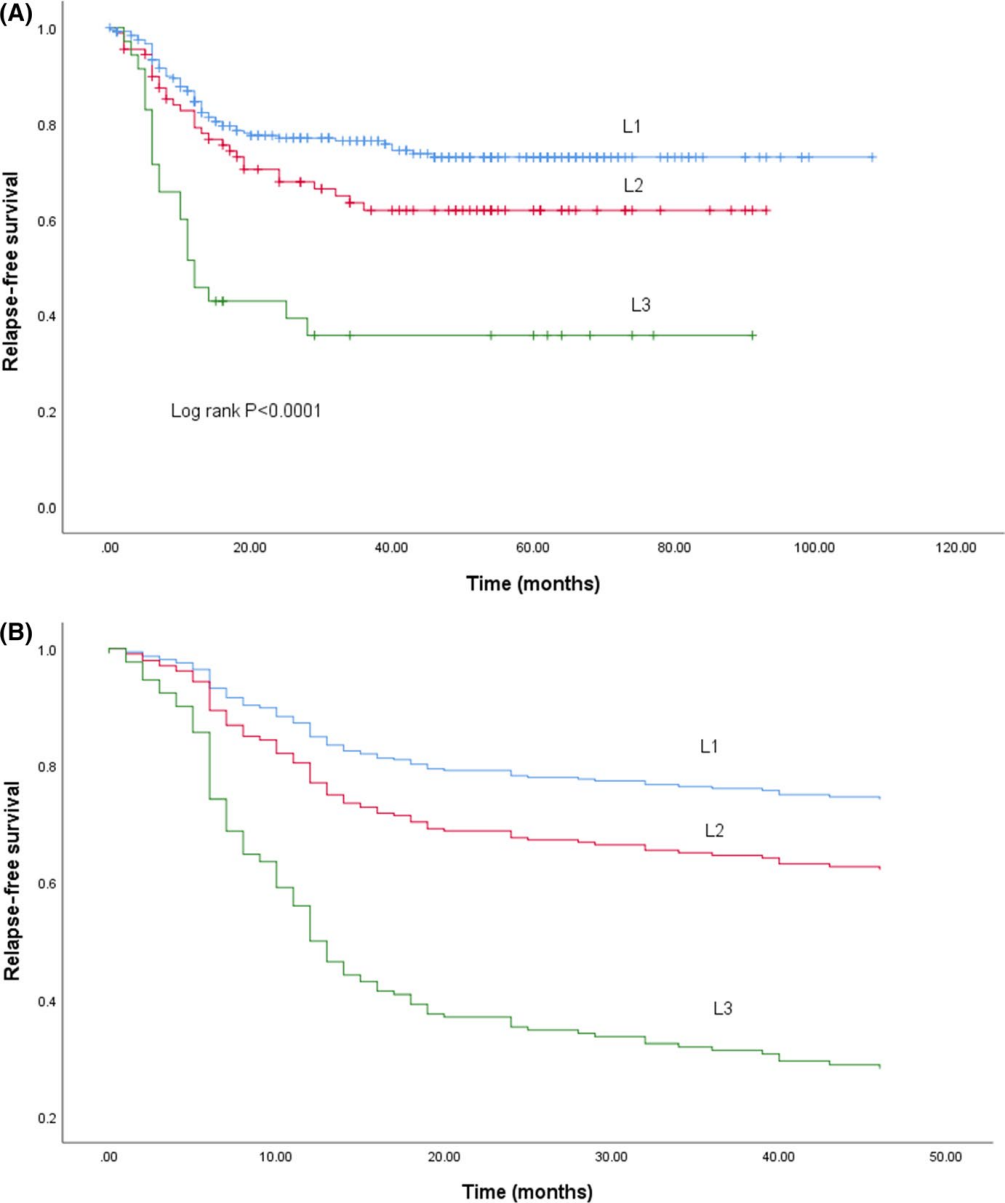
A, RFS among each L group (L1, L2, and L3). B, RFS among each L group (L1, L2, and L3) adjusting for KRAS status

Survival based on biomarker status is shown in Figure 2. KRAS wt patients showed better RFS (Figure 2A; *P* = .02), but BRAF status did not affect RFS (Figure 2B; *P* = .65). MSI‐H patients showed a better RFS compared with MSS (Figure 2C; *P* = .007). In a Cox multivariate model, the total number of LN invaded, KRAS mt, pathological venous invasion, elevated carcinoembryonic antigen (CEA) levels before surgery, and a skipped LN pattern were identified as poor prognostic factors. Overall, RFS of patients with a *sequential* metastatic LN pattern tended to be better compared with patients with a *skipped* pattern (Figure 3A; *P* = .086). No survival difference was observed between the sequential and skipped patterns in KRAS wt patients (Figure 3B; *P* = .82). Conversely, RFS of KRAS mt patients with a sequential pattern was better compared with those with a skipped pattern (Figure 3C; *P* = .018). Univariate analysis of RFS based on the maximal level of LN invasion showed a significant difference in RFS between L1, L2, and L3 (Figure 4A; *P* < .001). This difference remained unchanged after correction for KRAS status in a Cox model (Figure 4B; Table 3).

**TABLE 3:** Cox multivariate model for RFS

Variable	*P* value	Hazard ratio	95%CI	
			Lower	Upper
Total LN invaded	**.001**	1.071	1.027	1.117
KRAS	**.038**	1.555	1.025	2.358
BRAF	.765	0.879	0.380	2.038
Pathological lymphatic invasion				
0	.430	1		
1	.926	0.960	0.411	2.244
2	.955	0.981	0.508	1.896
3	.308	1.408	0.729	2.719
Pathological venous invasion				
0	**.036**	1		
1	**.030**	0.418	0.190	0.917
2	**.015**	0.484	0.270	0.868
3	.380	0.770	0.429	1.381
Elevated pre‐Op CEA	**<.001**	1.001	1.000	1.001
Skipped	**.020**	0.516	0.296	0.899

Bold values indicate *P* < .05 is considered as significant.

**FIGURE 5:**
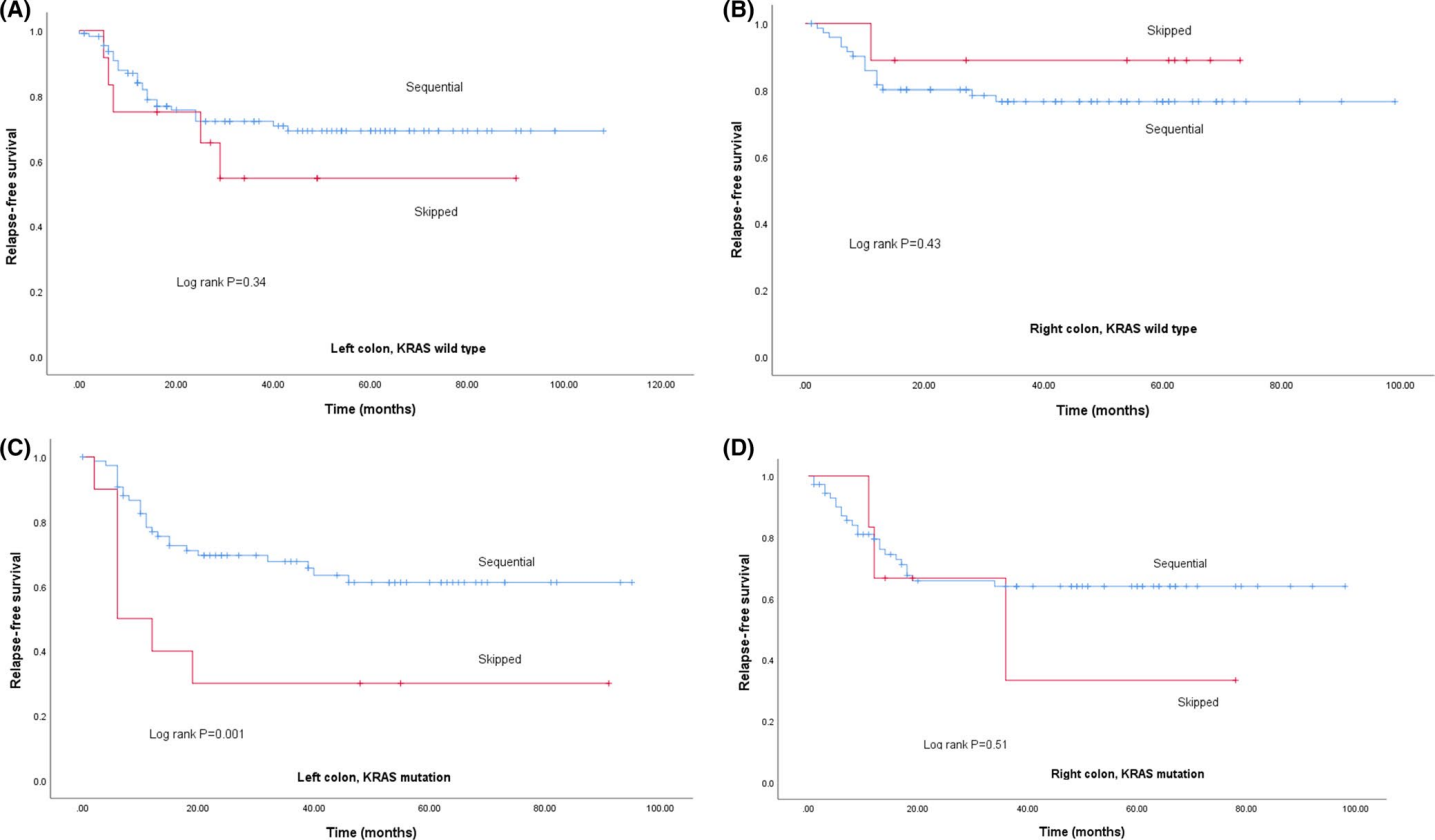
A, RFS of KRAS wt patients based on tumor sidedness (left colon; sequential = blue, skipped = red). B, RFS of KRAS wt patients based on tumor sidedness (right colon; sequential = blue, skipped = red). C, RFS of KRAS mt patients based on tumor sidedness (left colon; sequential = blue, skipped = red). D, RFS of KRAS mt patients based on tumor sidedness (right colon; sequential = blue, skipped = red)

Subgroup analysis based on tumor sidedness in KRAS wt and KRAS mt CC patients showed that in left‐sided CC, RFS of KRAS mt patients with a sequential pattern were better compared with those with a skipped pattern (Figure 5A (wt), *P* = .34 in left‐sided and Figure 5B (wt), *P* = .43 in right‐sided; Figure 5C (mt), *P* = .001 in left‐sided and Figure 5D (mt) *P* = .51 in right‐sided). Regarding BRAF, tumor sidedness, and LN skip pattern did not affect RFS. In MSS patients, RFS with a sequential anatomical spread pattern was better compared with those with a skipped pattern (*P* = .018), but there was no association of LN spread pattern with RFS in MSI‐H patients (*P* = .818).

## 4 DISCUSSION

Although the anatomical pathways of lymphatic spread in CC have been discussed for several decades, the exact mechanisms are not fully understood. Historically, 2 theoretical models have been advocated (Figure 1). In the Halsted model, lymphatic spread is a stepwise, orderly process in which cancer cells spread from the primary tumor to nearby LNs, then to intermediate nodes, subsequently to central nodes, and finally to distant organs such as the liver.^18^ The Halsted model is based on the assumptions that lymphatic progression can only arise when the previous LN station is breached, and that resection of all invaded nodes may result in a cure. Conversely, in the Fisher model, lymphatic as well as hematogenous metastasis occur early, and at random.^19^ When the Fisher model would apply to CC, extensive surgery to retrieve all invaded LN is unlikely to affect outcome. Recently, several phylogenetic analyses on a limited number of patients indicated that metastasis to distant organs and lymphatic spread occurred simultaneously in CC.^20^, ^21^, ^22^ We have previously reported the patterns of LN spread in stage III CC, but molecular data were not incorporated.^8^, ^20^ It is likely that the temporal and anatomical patterns of lymphatic spread are governed by the molecular and genetic properties of the primary cancer. This is the first analysis of the association between the anatomical pattern of LN spread and selected molecular biomarkers in CC.

The results of the present study confirmed that the pattern of LN metastasis in stage III CC is related to biomolecular properties. First, we found striking differences in the proportion of patients with a *skipped* metastatic pattern in accordance with MSI and BRAF status. In the entire cohort, the proportion of patients with a skipped metastatic LN pattern was comparable with previous studies, ranging between 0% and 18%.^8^, ^9^, ^23^ Patients with a deficient mismatch repair had a much lower proportion of skipped LN stations (4% vs 10.5%), although this difference was not statistically significant due to the relatively small number of MSI‐H patients. Previous studies have shown that mismatch repair deficient tumors are characterized by extensive infiltration by activated T‐cells.^24^, ^25^, ^26^, ^27^, ^28^, ^29^, ^30^ Little information is known on the effect of mismatch repair (MMR) status on the microenvironment of locoregional LN. One study found that, compared with MSS, LN from MSI‐H tumors tended to have more follicular hyperplasia and very prominent paracortical hyperplasia.^31^ As the paracortex is a T cell zone, this phenotype may reflect regional infiltration of T lymphocytes, which may prevent metastatic cells to effectively transfer node station and cause a skipped metastatic LN pattern. Similarly, we found that patients harboring a BRAF mutation were significantly *less* likely to display a skipped metastatic nodal pattern (9.3% vs 20%). This finding may be related to the fact that a subset of patients with BRAF mutations is known to display a high stromal CD8‐positive cell infiltration and to have a prognosis that is similar to wt patients. This is supported by the fact that we did not find a survival difference in accordance with BRAF mutational status, in contrast with other published reports. Therefore, our findings may not be applicable to all patients with a BRAF mutation.

In addition, we found that the total number of invaded LN in MSI‐H patients was lower compared with those with MSS tumors. Belt et al have reported that a higher number of LN harvested was associated with MSI status but the association of the number of LN invaded with MSI status was not mentioned.^32^ In our study, the number of LN harvested is similar in MSS and MSI‐H tumors. We have also advocated the prognostic significance of positive central lymph node (L3) in our previous studies.^8^ In our series, there are only 2 MSI‐H patients with invaded central LN, following a sequential pattern, and no recurrence was observed. Although the sample size is small, these findings suggest that MSI‐H patients may benefit more from extensive lymphadenectomy compared with MSS patients.

Regarding the impact of LN spread pattern and biological status on survival, RFS of CC patients with a sequential spread pattern was longer compared with those with a skipped pattern. In subgroup analysis based on KRAS status, there was no difference in RFS among KRAS wt CC patients between sequential pattern and skipped patterns. However, in KRAS mt patients, RFS of patients with a sequential pattern was longer compared with those with a skipped pattern. When patients were classified based on the location of invaded LN (L1, L2, L3), KRAS status did affect the survival outcome in each L group. When patients were divided into right vs left colon cancer, RFS was shorter in left‐sided KRAS mt CC with a skipped pattern, but no difference in RFS was observed in right‐sided KRAS mt CC with a skipped pattern. Large cohort analysis showed that RFS was longer in left‐sided compared with in right‐sided stage III CC.^33^ Guinney et al proposed CMS, by which colorectal cancer was classified into 4 molecular subtypes. CMS3 is characterized by frequent KRAS mutations, chromosomal instability, and marked WNT and MYC signaling activation.^34^ CMS1 encompasses the majority of MSI tumors, including hypermutated and hypermethylated subtypes. From the combined analysis of CALGB/SWOG80405 and FIRE‐3 carried out by Aderka et al, overall survival of patients with CMS3, which accounted for 13% of all the left‐sided CC, was worse compared with that of patients with CMS2 and CMS4, which accounted for 78%.^35^ Further analysis using other gene mutations may help to clarify the mechanism to cause skip metastases of LN.

The survival benefit of adjuvant chemotherapy in stage III CC patients with MSI‐H is still under discussion.^36^ The pooled analysis of 2 phase III trials (NCCTG N0147 and PETACC8) indicated a small survival benefit of adjuvant FOLFOX in MSI‐H tumors.^37^ The pooled data of the IDEA collaboration showed that almost half of the patients who received 6 mo of FOLFOX or CapOx chemotherapy experienced grade 2 or greater neurotoxicity.^38^ As the benefit of 5‐FU monotherapy seems small in stage III CC with MSI‐H from the pooled analysis of 5 RCTS,^39^ short cycles of FOLFOX or CapOx until the onset of severe neurotoxicity can be an option for this population.

There are several limitations and uncertainties in our study. First, this is a retrospective study and the sample size of MSI‐H and BRAF mt patients was small. Additionally, RFS is comparatively high with few events, therefore statistical power of the analyses was limited. As measurement of MSI‐H and BRAF mt for stage III CC is now covered by health insurance, future larger scale studies can be foreseen. Second, NRAS status was lacking in 171/369 patients, therefore analysis stratified into NRAS status was not performed. Third, the definition of “skipped” and “sequential” was based on the distribution pattern of LN invaded, which was just evaluated microscopically using 1 slice of the specimen. Yamamoto et al evaluated the presence of micrometastases in negative LN, evaluating CEA mRNA extracted from these LNs by RT‐PCR.^40^ In total 24% of the stage II CC patients were CEA mRNA positive, indicating possible micrometastases in negative LN. Our group is now planning the re‐evaluation of LN spreading pattern using OSNA.^41^

In conclusion, the pattern of invaded LN varies in accordance with molecular status. ‘skipped’ LN metastases were twice as frequent in BRAF wt and MSS tumors, compared with BRAF mt and MSI tumors. Less intensive treatment strategies, including less extensive lymphadenectomy, may be an option in MSI‐H CC because the LN spread pattern is mostly sequential, and less LN are usually invaded. Further studies in larger datasets are warranted to confirm our findings.

## CONFLICT OF INTEREST

The authors have no conflicts of interest to disclose.
